# 827. Meek Micrografting for Full-thickness Hand Burns in High TBSA Burn Patients

**DOI:** 10.1093/jbcr/irag033.266

**Published:** 2026-04-06

**Authors:** Amanda Seymour, Chinenye O Ogbonnah, Anirudh Sudarshan, Brent Egeland

**Affiliations:** University of Texas at Austin Dell Medical School, Austin, TX; University of Texas at Austin Dell Medical School, Austin, TX; University of Texas at Austin Dell Medical School, Austin, TX; University of Texas at Austin Dell Medical School, Austin, TX

## Abstract

**Introduction:**

Full-thickness hand burns are uniquely challenging. Split-thickness sheet skin grafting is the current gold standard, however, requires large donor sites – impractical or impossible in high total body surface area (TBSA) burns. Additionally, necessary immobilization to prevent shear introduces rehabilitation challenges. Meek micrografting, comprised of many discontinuous, several-millimeter skin squares, theoretically allows near-immediate motion in any direction, easily conforms to complex shapes, and can be expanded in up to a 9:1 ratio. We hypothesized Meek grafting would allow earlier hand motion while maximizing use of little available harvest sites in high TBSA burn patients.

**Methods:**

Meek micrografting to the hand was performed in seven high TBSA burn patients presenting to our academic Level 1 trauma and burn center. Burn characteristics, graft take, time of therapy initiation, and range of motion were recorded.

**Results:**

Five males and two females average full-thickness TBSA of 32.2% (range 4% to 56%) with hand involvement were treated with Meek micrografting. Average hand burn dimension was 125 cm2. Three 2:1 ratio meek grafts were used per hand. Average donor site was 54 cm2. Average graft take at evaluation five days postoperatively was >95%. Compared to sheet grafting, mobility initiation was reduced from 10 to 5 days. Long-term (>6 month) range of motion, patient reported outcomes, and revision rates were equivalent to sheet grafting.

**Conclusions:**

Meek micrografting is an effective treatment method for full-thickness hand burns. Compared to sheet grafting, the technique reduces donor site requirements, allows early therapy, and long-term functional outcomes appear equivalent.

**Applicability of Research to Practice:**

Preliminary results demonstrate that Meek micrografting is a safe, effective, and possibly superior coverage method for hand burns in the high TBSA patient.

**Funding for the study:**

N/A.

